# Seeing your own or someone else's hand moving in accordance with your action: The neural interaction of agency and hand identity

**DOI:** 10.1002/hbm.24958

**Published:** 2020-02-24

**Authors:** Lukas Uhlmann, Mareike Pazen, Bianca M. van Kemenade, Olaf Steinsträter, Laurence R. Harris, Tilo Kircher, Benjamin Straube

**Affiliations:** ^1^ Department of Psychiatry and Psychotherapy University of Marburg Marburg Germany; ^2^ Center for Mind, Brain and Behavior (CMBB) University of Marburg Marburg Germany; ^3^ Core Facility Brain Imaging University of Marburg Marburg Germany; ^4^ Department of Psychology York University Toronto Ontario Canada

**Keywords:** agency, fMRI, forward model, hand identity, hand movement, prediction, self‐other

## Abstract

Forward models can predict sensory consequences of self‐action, which is reflected by less neural processing for actively than passively generated sensory inputs (BOLD suppression effect). However, it remains open whether forward models take the identity of a moving body part into account when predicting the sensory consequences of an action. In the current study, fMRI was used to investigate the neural correlates of active and passive hand movements during which participants saw either an on‐line display of their own hand or someone else's hand moving in accordance with their movement. Participants had to detect delays (0–417 ms) between their movement and the displays. Analyses revealed reduced activation in sensory areas and higher delay detection thresholds for active versus passive movements. Furthermore, there was increased activation in the hippocampus, the amygdala, and the middle temporal gyrus when someone else's hand was seen. Most importantly, in posterior parietal (angular gyrus and precuneus), frontal (middle, superior, and medial frontal gyrus), and temporal (middle temporal gyrus) regions, suppression for actively versus passively generated feedback was stronger when participants were viewing their own compared to someone else's hand. Our results suggest that forward models can take hand identity into account when predicting sensory action consequences.

## 1 INTRODUCTION

In order to efficiently react to changes in our environment, sensory events caused by one's own actions need to be distinguished from sensory events caused by other agents (Cullen, 2004). This is not a trivial task, since sensory receptors are similarly activated irrespective of what caused a given stimulus (Crapse & Sommer, 2008). However, a pivotal difference between actively and passively generated sensory input is that the timing and intensity of the former can be predicted using a neural signal reflecting a copy of the motor command (efference copy; Elijah, Le Pelley, & Whitford, 2016; Pynn & Desouza, 2013; Shergill, Samson, Bays, Frith, & Wolpert, 2005; Sperry & Stone, 1950; von Holst & Mittelstaedt, 1950). Efference copies are used by internal forward models to predict the sensory outcome of the planned action (Blakemore, Wolpert, & Frith, 2000; Miall & Wolpert, 1996; Wolpert, Ghahramani, & Jordan, 1995). It has been shown that forward models influence the perception of the sensory consequences of one's own actions: Accurately predicted sensory input is canceled out from further processing, enabling the system to focus on potentially relevant, unpredicted stimuli (Bays, Flanagan, & Wolpert, 2006; Wolpert & Flanagan, 2001). In line with this, it has been demonstrated that actively relative to passively generated sensory input is associated with less neural processing (suppression effect), in auditory (e.g., Heschl's gyrus), somatosensory (e.g., postcentral gyrus), and visual (e.g., calcarine sulcus) areas for discrete actions (Blakemore, Wolpert, & Frith, 1998; Straube et al., 2017). Similar effects were reported in visual and somatosensory cortices as well as the right posterior superior temporal sulcus for continuous action feedback such as videos of a moving hand (Arikan et al., 2019; Limanowski, Sarasso, & Blankenburg, 2018; Pazen et al., 2020).

Moreover, the outcome of the comparison between sensory predictions and reafferent feedback serves as a cue for the attribution of authorship over a movement and its sensory consequences (sense of agency), with greater mismatches (prediction errors) indicating a higher likelihood that the movement was externally produced (Sato & Yasuda, 2005; Wolpert & Flanagan, 2001). Perturbed agency (e.g., as assessed by comparing passive with active movements), has been linked to the temporo‐parietal junction, frontal regions (e.g., superior frontal gyrus and medial frontal gyrus) and the cerebellum (Blakemore, Oakley, & Frith, 2003; Tsakiris, Longo, & Haggard, 2010). Furthermore, temporal mismatches between action and sensory outcome are associated to activation in posterior parietal regions, such as the superior parietal lobule (Leube, Knoblich, Erb, & Kircher, 2003), the precuneus (Farrer et al., 2004; Farrer & Frith, 2002), and the temporo‐parietal junction (Leube, Knoblich, Erb, Grodd, et al., 2003; Leube, Knoblich, Erb, Schlotterbeck, & Kircher, 2010; Limanowski, Kirilina, & Blankenburg, 2017; van Kemenade et al., 2019). Finally, activation in the temporo‐parietal junction, and especially in the angular gyrus, is associated with explicit awareness of non‐agency, that is, the subjective experience of *not* having caused a sensory event (Farrer et al., 2008; Hughes, 2018).

It is still an open question, however, whether these neural correlates of the perception of one's own action consequences are influenced by body identity, that is, by visual features that determine if a seen body part belongs to oneself or not. If so, the extent of neural suppression would differ dependent on whether one's own or someone else's hand is seen. Specifically, sensory feedback would become more (own hand) or less (someone else's hand) predictable, which would be associated with decreased or increased prediction error processing in posterior parietal regions, respectively. Incorporating body identity into forward models would ultimately aid the brain to efficiently monitor the source of sensory feedback in social situations (e.g., when dancing or shaking hands with another person), for instance by providing highly learned default predictions for action consequences involving one's own body.

Previous studies have revealed that visual resemblance of a seen body part with one's actual body aids embodiment of fake hands (Bertamini & O'Sullivan, 2014; Haans, Ijsselsteijn, & de Kort, 2008; Tsakiris, Carpenter, James, & Fotopoulou, 2010) or virtual bodies (Gonzalez‐Franco & Peck, 2018; Waltemate, Gall, Roth, Botsch, & Latoschik, 2018). For instance, in the rubber hand illusion (RHI, Botvinick & Cohen, 1998), one's right hand (which is obstructed from view) and a fake rubber hand are synchronously stroked, leading to the experience that the rubber hand becomes a part of one's own body (body ownership). Ownership is usually assessed by explicit (e.g., questionnaires) or implicit measures (e.g., changes in the temperature, electrodermal activity or perceived location of the real hand; for a review, see Kilteni, Maselli, Kording, & Slater, 2015). Crucially, while studies using the RHI have revealed that the sense of agency can be established in the absence of body ownership (Kalckert & Ehrsson, 2012, 2014), a recent study has shown that sensory suppression increases in the presence of ownership over the rubber hand (Kilteni & Ehrsson, 2017a). Moreover, after having embodied a rubber hand, suppression of sensory feedback caused by one's actual hand is diminished, suggesting that body ownership determines sensory suppression in the presence of agency (Kilteni & Ehrsson, 2017a). Therefore, forward models seem to form predictions specific for the embodied hand, be it one's own or a fake one (Aymerich‐Franch, Petit, Kheddar, & Ganesh, 2016). It has been demonstrated that body ownership can produce sensory suppression even in the absence of efferent signals, underpinning the pivotal role of body representation in sensory processing (Burin, Pyasik, Salatino, & Pia, 2017; Pyasik et al., 2019).

While previously mentioned studies have revealed a strong link between body ownership and sensory suppression, the role of body identity in predictive processing remains elusive. Although body identity and body ownership appear similar at first, the concepts are still distinct: A genuine experience of body ownership requires temporal congruence between visual, somatosensory, and proprioceptive signals (for a review, see Braun et al., 2018), whereas the identity of a body part can be inferred by unisensory (e.g., visual) input alone, for instance when seeing oneself in a photograph or in a mirror (Conson, Aromino, & Trojano, 2010; Preston, Kuper‐Smith, & Ehrsson, 2015; Rice, Phillips, Natu, An, & O'Toole, 2013). Yet, visual appearance of one's body offers strong cues to distinguish between self and other and has thus been considered an integral component of the corporeal self (Faccio, 2013; Frassinetti et al., 2009; Kruse, Bogler, Haynes, & Schütz‐Bosbach, 2016; Myers & Sowden, 2008). Therefore, in the current study, we investigated whether the identity of a seen body part influences the neural processing of actively and passively generated sensory action consequences.

To let participants focus on the sensory consequences of their actions, delay detection tasks have proven useful (e.g., Hoover & Harris, 2012; Leube et al., 2010; Leube, Knoblich, Erb, Grodd, et al., 2003; Leube, Knoblich, Erb, & Kircher, 2003; Pazen et al., 2020; Straube et al., 2017; van Kemenade, Arikan, Kircher, & Straube, 2016). Participants have to detect temporal action‐feedback asynchronies, with the advantage that delays can be applied to different kinds of feedback (e.g., different hands). Previous studies investigating delay detection performances during active and passive movements have mostly used button presses and discrete action outcomes, revealing enhanced performances for actively generated feedback (Shimada, Hiraki, & Oda, 2005; van Kemenade et al., 2016). In contrast, for feedback displaying continuous hand movements, delay detection performances are decreased for active compared to passive movements, indicating that perceptual suppression impairs the comparison between continuous actions and feedback in the temporal domain (Arikan et al., 2019; Pazen et al., 2020; van Kemenade et al., 2019). Furthermore, delay detection tasks have been used to investigate the influence of hand appearance on the perception of action feedback (Hoover & Harris, 2012, 2015; Zopf, Friedman, & Williams, 2015). Overall, delay detection tasks are suited to investigate the neural correlates of the perception of sensory action consequences.

In the current study, functional magnetic resonance imaging (fMRI) was used to measure brain activation while participants were seeing their own or someone else's hand moving in accordance with their own action. To assess the role of forward models in perceiving the sensory consequences of one's own actions, we used a custom‐made MR‐compatible movement device, which could be moved actively by the participants or passively by air pressure (Pazen et al., 2020). Participants had to detect delays (0–417 ms) that were inserted between movements and the visual feedback. This design allowed us to investigate the contributions of agency (active vs. passive movements) and hand identity (“self” vs. “other” feedback) to the perception of sensory action consequences. Based on the literature, we expected a suppression effect reflected by less BOLD activation in sensory areas (Arikan et al., 2019; Blakemore et al., 1998; Limanowski et al., 2018; Pazen et al., 2020; Straube et al., 2017) and worse delay detection performances (Arikan et al., 2019; Pazen et al., 2020; van Kemenade et al., 2019) during active than passive movements. Moreover, we assumed that forward models take hand identity into account when predicting the sensory consequences of one's own action. The comparison of active compared to passive conditions should thus reveal stronger suppression (as indicated by a reduction of neural activation [BOLD suppression] in sensory areas and higher behavioral detection thresholds for active trials) if “self” (but not “other”) feedback is presented. This interaction effect was also expected to be observed in brain regions related to agency and prediction error processing, such as posterior parietal regions.

## 2 MATERIALS AND METHODS

### 2.1 Participants

Twenty‐four right‐handed healthy participants (13 female, age: 20–35 years, mean age: 26.62, *SD*: 4.01 years) with normal or corrected‐to‐normal vision took part in the experiment. One participant did not detect any delay in the “active self” condition and was thus excluded from further analyses (for further details, see Experimental design). Thus, the final sample consisted of 23 participants (12 female, age: 20–35 years, mean age: 26.43 years, *SD*: 3.99 years). Right‐handedness was confirmed by the Edinburgh Handedness Inventory (Oldfield, 1971). All participants gave informed consent and were remunerated for their participation. The experiment was approved by the local ethics committee and performed in accordance with the Declaration of Helsinki.

### 2.2 Stimuli and equipment

**Figure 1:**
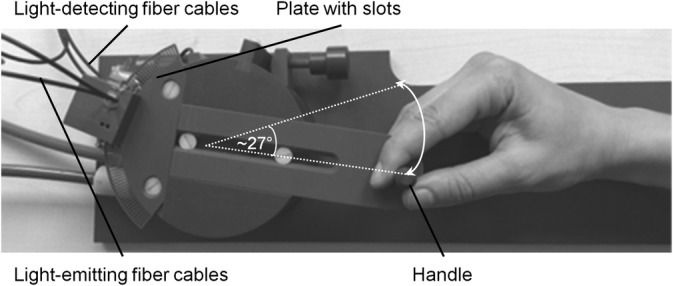
Movement device. During the experiment, participants held the handle of a device to perform movements. The handle could be moved along a circular arc through an angle of approximately 27°. The device could be moved actively by the participant or passively by air pressure. The hand is shown here in the “neutral position.” In the fMRI session, the right arm was stretched out parallel to the participant's leg with the palm of the right hand facing the right outer thigh. To monitor movements, a plate with slots as well as optic fiber cables were attached to the device (see text for further details on the motion detection algorithm). Note that during the experiment, the plate was covered by a box and is visible here for illustration purposes only

In the experiment, participants performed hand movements while holding the handle of a custom‐made device (see Figure 1). The device could be moved from the left (neutral position) to the right and back again along a circular arc (central angle: ~27°; trajectory: about 5 cm). Movements could be actively generated by the participant or the hand could be passively moved by the device. Air pressure was used to move the device in passive conditions. To monitor speed and direction of movements during the experiment, a circular plate containing slots was attached to the movement device. Light‐emitting and light‐detecting fiber cables were placed perpendicular to the slot plate. When the device was moved, the light signal (from the emitting cables) was interrupted by the slot plate. This interruption was captured by the light‐detecting cables, thereby providing information on both the position of the movement device's handle within ~3′ (~1 mm) on the circular arc, and the direction of the movement.

**Figure 2:**
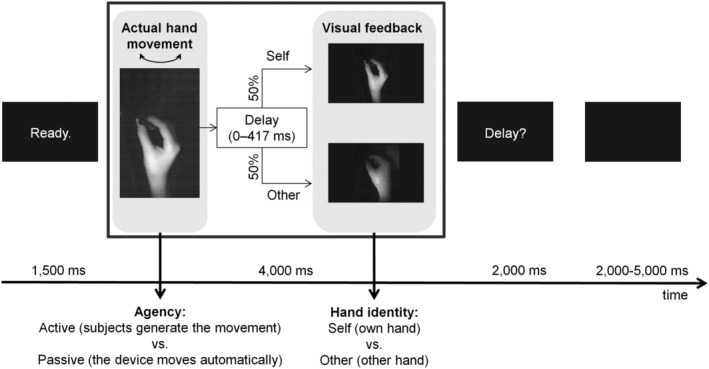
Experimental paradigm. The beginning of each trial was indicated by “Ready.” on the screen. Thereafter, the participant's own hand or someone else's hand was displayed, and movements could be performed. Movements could either be generated by the participant (“active” condition) or by the device using air pressure (“passive” condition). Videos were either presented in real time or delayed. Subsequently, a question (“Delay?”) appeared on the screen, indicating that participants could now report whether they detected a delay or not. At the end of each trial, the screen turned black for a variable inter‐trial interval. A video demonstration of the experimental paradigm (outside of the MR scanner, for illustrative purposes) is available in the supporting information (Video S1) and at: http://doi.org/10.5281/zenodo.2621302

On 50% of the trials (“self” trials), the participant's own hand was recorded with a high‐speed camera (MRC High Speed, MRC Systems GmbH, Heidelberg, Germany; refresh rate: 4 ms) and played onto a computer screen (refresh rate: 60 Hz). In the other 50% of the trials (“other” trials), a previously recorded hand of another person holding the movement device was displayed. All images were displayed in first‐person perspective (see Figure 2). Prior to the fMRI experiment, the camera was adjusted for each participant individually so that the participant's hand was positioned in the middle of the screen (see Figure 2). In some cases, participants' clothing was visible. To increase discriminability between “self” and “other” trials, the “other” hand was from a person of the opposite sex to the participant. Importantly, the movement of the displayed “other” hand was directly coupled to the actual hand movement (e.g., if the movement device was moved to the right, the “other” hand moved to the right as well). To achieve this, each position of the handle (i.e., slot number) called a frame of the previously recorded “other” hand at the same position, resulting in a coherently displayed movement of the “other” hand when the device was moved. We then randomly inserted variable delays (0, 83, 167, 250, 333, or 417 ms + internal setup delay of 43 ms, respectively) between the onset of the actual and the displayed movement (both “self” and “other”). These delays corresponded to multiples of frames determined by the screen's refresh rate (0, 5, 10, 15, 20, and 25 frames at 60 Hz). The setup was controlled by custom‐written software on a personal computer (Intel® Core™ i5‐4,570 CPU, 3.20 GHz, 4 GB RAM, AMD Radeon HD8570D Graphics Card, 32‐bit operating system, Windows 7 Professional [Microsoft Corporation, 2009]). Due to scanner noise, setup‐related acoustic signals were inaudible during the experiment.

### 2.3 Experimental design

A within‐subjects design with the factors agency (active vs. passive) and hand identity (“self” vs. “other”) was used, resulting in four conditions: active self, passive self, active other, passive other. Before participants took part in the fMRI session, they completed a preparatory session in which they were familiarized with the setup (interval between preparatory and scanning session: 5–19 days, mean interval ± *SD*: 11.61 ± 4.01 days). The same experimental paradigm was used in both sessions (see Figure 2), with the difference that only one run was carried out in the preparatory session, while two runs were carried out in the fMRI session.

Each run contained 48 trials (overall duration: ~9 min per run) and was divided into an active and a passive block. Within each block, “self” and “other” hand feedback occurred intermingled and in randomized order. At the beginning of each block, a cue (“Active” or “Passive”) was displayed for 4,000 ms, indicating whether the movements were to be initiated actively by the participant or passively by the movement device. Note that all cues were presented in German. Trials started whenever “Ready.” was displayed on the screen (duration: 1,500 ms). Thereafter, the participant's own hand or the “other” hand was displayed for 4,000 ms. In active blocks, participants were instructed to execute the movement at any point during the time a hand (“self” or “other”) was visible. In passive blocks, movement onset was programmed to start 500 ms after stimulus onset. Subsequently, “Delay?” was displayed for 2,000 ms, signaling participants to respond via button press whether they perceived a delay between action and feedback or not. Within each condition, each of the six delays occurred twice per run. At the end of each trial, the screen turned black for a variable duration (intertrial intervals: 2,000, 3,000, 4,000, or 5,000 ms). In total, trials lasted between 9,500 to 12,500 ms. The reported analyses were thus based on 96 trials distributed across two runs (i.e., 24 trials per condition).

### 2.4 Procedure

During the preparatory session, participants were seated in front of a computer screen with their right hand holding the movement device. Participants held the movement device such that the upper part of the handle was held by the index finger and the thumb, with the remaining fingers of the right hand touching the lower part of the grip (see Figure 1). Participants' right hands were obstructed from view by a curtain. All instructions were given orally in German. Participants were instructed to perform hand movements by extending and subsequently returning their wrist back to a neutral wrist position while holding the handle. To minimize differences in movement duration between active and passive conditions, participants were asked to complete movements in about 1.5 s, which was checked using a metronome and corrected if necessary. In passive blocks, they were instructed to relax their wrist so that the hand could be moved by the device. A paired *t*‐test showed no significant differences in movement durations between active and passive movements, *t*(22) = 0.214, *p* = .833, *d* = 0.05 (active: *M* = 1,228 ms, *SEM* = 29 ms; passive: *M* = 1,239 ms, *SEM* = 45 ms). Next, participants were informed that their movements would be recorded with a camera and displayed on the screen and that sometimes someone else's hand would be displayed. Importantly, participants knew that the movement of the seen hand was always coupled with their actual hand movement, regardless of whether their own or the “other” hand was displayed. Finally, they were told that the displayed movement could be delayed relative to the actual movement. Participants were instructed to respond whether they perceived a delay or not by pressing one of two keys with their left middle or index finger (button assignment counterbalanced across participants). Before participants completed the preparatory run, a short training consisting of eight trials (two per condition with either a 0 ms or 417 ms delay) was carried out. During this training, they received visual feedback (“Yes” or “No”) after each trial on whether there was a delay or not.

In the fMRI session, participants lay supine in the MRI‐scanner, with their right arm stretched out parallel to their body so that the movement device (placed next to the right thigh) could be reached comfortably. The visual feedback was displayed on a screen behind the MRI tube. Participants looked upward and saw the screen reflected in a tilted mirror mounted above their head. Foam pads were used to reduce head motion during scanning.

After the scanning session (i.e., outside the scanner room), participants were asked how strongly each of the displayed hands felt like their own hand to ensure that our manipulation of hand identity was successful. The question could be answered on a 10‐point‐Likert scale ranging from 1 (“very weak”) to 10 (“very strong”). A paired *t*‐test revealed that the displayed hand felt more like their own hand during “self” compared to “other” trials (“self”: *M* = 7.61, *SEM* = 0.52; “other”: *M* = 4.83, *SEM* = 0.60), *t*(22) = 3.959, *p* = .001, *d* = 0.83. Further post‐experiment questions on hand identity are listed in the Supplement (Table S1).

### 2.5 Functional data acquisition

To acquire functional MRI data, a 3 Tesla Magnetom Trio Tim scanner (Siemens, Erlangen, Germany) and a 12‐channel head‐coil were used. A T2*‐weighted gradient‐echo echoplanar imaging sequence was applied (repetition time [TR]: 1,650 ms, echo time [TE]: 25 ms, flip angle: 70°). During each run, 330 volumes were obtained, each covering the brain in 34 axial slices (matrix: 64 × 64, field of view [FoV]: 192 mm × 192 mm, slice thickness: 4 mm, voxel size: 3 mm × 3 mm × 4.6 mm [including a gap of 0.6 mm]), which were acquired in descending order. A T1‐weighted MPRAGE sequence (TR: 1,900 ms, TE: 2.26 ms, flip angle: 9°) was applied to record anatomical images (matrix: 256 × 256, FoV: 256 mm × 256 mm, slice thickness: 1 mm, voxel size: 1 mm × 1 mm × 1.5 mm [including a gap of 0.5 mm]).

### 2.6 Behavioral data analysis

All trials in which no movement or no response was registered were excluded from analysis (0.8% of all trials). For each subject, the proportion of “yes” responses was calculated for each delay in each condition. Cumulative normal functions were then fit to the data using Psignifit4 (Schütt, Harmeling, Macke, & Wichmann, 2016) in MATLAB 7.9 (The Mathworks Inc., 2009). From these functions, detection thresholds (i.e., delays at which the probability for “yes” responses was .50, based on the fit psychometric function) and corresponding slopes (standard deviations) were derived and entered into separate factorial repeated‐measures ANOVAs implemented in SPSS24 (IBM SPSS Statistics, Chicago, IL). A sensitivity analysis run in GPower 3.1 (Faul, Erdfelder, Buchner, & Lang, 2009) revealed that the required effect size to detect a statistically significant effect was *f* = 0.25 (*ω*_*p*_^2^ = 0.07; given *α* = .05, *β* = .80, and *N* = 23).

### 2.7 Functional and anatomical data preprocessing and analysis

The analyses of functional data were performed using standard procedures of Statistical Parametric Mapping (SPM12, Wellcome Trust Centre for Neuroimaging, University College London, UK) in MATLAB 7.9 (The Mathworks Inc., 2009). First, functional images were realigned to correct for head motion. Runs in which the translation exceeded 3 mm were excluded from further analyses (*n* = 1). Each participant's anatomical image was coregistered to their first functional image and then segmented. The deformation field calculated in the segmentation step was used to spatially normalize the functional images to the Montreal Neurological Institute (MNI) standard space, resampled to a voxel size of 2 mm × 2 mm × 2 mm. Finally, the functional images were smoothed with an 8 mm × 8 mm × 8 mm full width at half‐maximum Gaussian kernel. Subsequently, the functional data were analyzed using the general linear model (GLM). For each participant, regressors of interest modeling the blood oxygenation level‐dependent (BOLD) signal during the time a hand was displayed on the screen were defined for each experimental condition (i.e., active self, passive self, active other, passive other). Additionally, six motion parameters as well as regressors modeling the BOLD signal during the presentation of cues and delay questions were included as regressors of no interest. All regressors were convolved with the canonical hemodynamic response function (HRF). For each voxel, a 128 s high‐pass filter was applied to remove low‐frequency noise from the time series. *T*‐maps were calculated by separately contrasting each regressor of interest against an implicit baseline. To analyze group effects, the contrasts estimates obtained from each subject were entered into a flexible factorial model.

Based on our hypotheses, we expected a downregulation of neural activation (i.e., suppression) during active compared to passive trials independent of hand identity, as assessed in a *t‐*contrast [(passive self + passive other) > (active self + active other)]. An additional *t‐*contrast was used to investigate increased processing involved in distinguishing “other” from “self” hand feedback independent of agency [(active other + passive other) > (active self + passive self)]. For completeness, *t‐*contrasts exploring the opposite main effects of agency [(active self + active other) > (passive self + passive other)] and hand identity [(active self + passive self) > (active other + passive other)] are reported as well. Finally, we focused on differences in BOLD suppression for active versus passive movements depending on hand identity (i.e., agency × hand identity interaction effect). We expected larger neural suppression of actively generated feedback during “self” than “other” trials, as reflected in an interaction *t‐*contrast [(passive self > active self) > (passive other > active other)].

A Monte‐Carlo simulation with 10,000 iterations was run to obtain a minimum cluster size that ensures correction for multiple comparisons at *p* < .05, assuming an individual voxel type I error of *p* = .001, uncorrected for multiple comparisons (Slotnick, 2017; Slotnick, Moo, Segal, & Hart, 2003). Applying the estimated smoothness of the functional data (13.5 mm), a cluster extent threshold of 83 resampled voxels was obtained. The corresponding height threshold at the whole brain level was *T* = 3.19. Only clusters that exceed this threshold will be reported. Locations of significant activations were labeled using the automated anatomical labeling (AAL) application (Tzourio‐Mazoyer et al., 2002) implemented in SPM12.

## 3 RESULTS

### 3.1 Behavioral results

**Figure 3:**
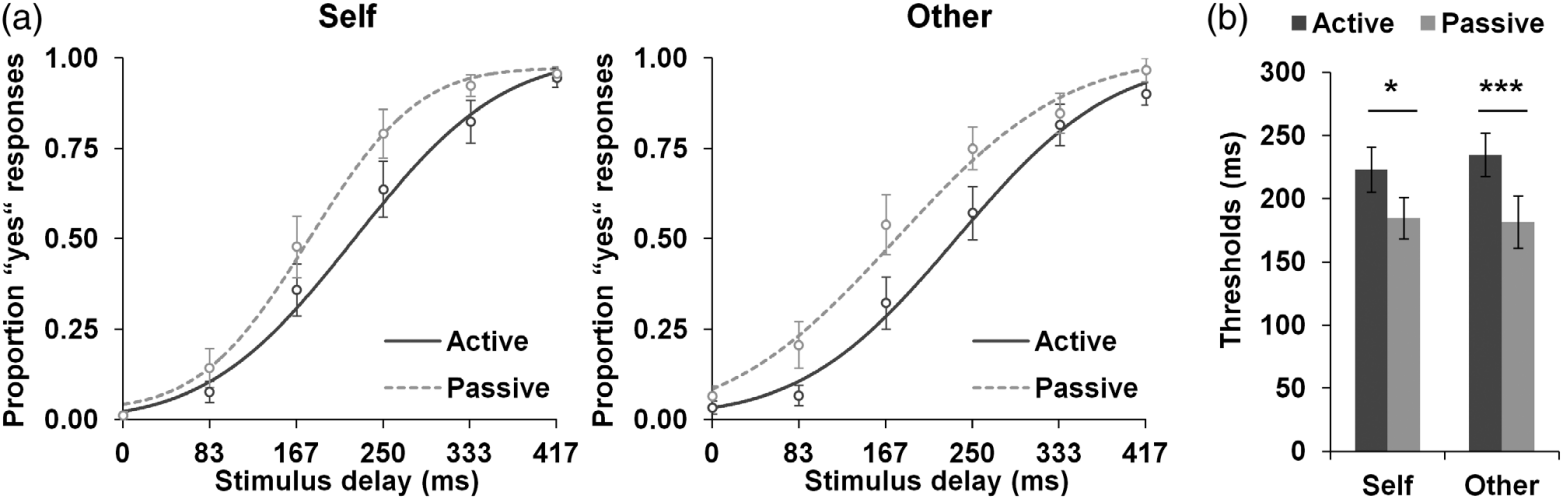
Behavioral results. (a) Group psychometric functions (*N* = 23) for all conditions. Psychometric functions were fit on averaged delay detection data for illustration purposes only. Statistical analyses were performed on individual subject data. (b) Mean thresholds (delays at which the probability for “yes” responses regarding the presence of a delay was .50, based on the fit psychometric function) for all conditions. Delay detection performance was significantly worse during active compared to passive trials across “self” and “other” conditions, as indicated by longer delays needed for detection. *p < .05; ***p < .001. Error bars show standard errors of the means (*SEM*s). Note that an internal setup delay of 43 ms must be added to all delay values

Group psychometric functions are displayed in Figure 3a. Thresholds and slopes derived from individual subject psychometric functions were entered into repeated‐measures ANOVAs with the factors agency (active vs. passive) and hand identity (“self” vs. “other”). The analysis of thresholds revealed a main effect of agency, *F*(1, 22) = 20.762, *p* < .001, *ω*_*p*_^2^ = 0.452, with higher thresholds (i.e., longer delays needed for detection) for active (*M* = 229 ms, *SEM* = 16 ms) than for passive (*M* = 183 ms, *SEM* = 18 ms) movements. This effect was significant during both “self” trials, *F*(1, 22) = 7.524, *p* = .012, *ω*_*p*_^2^ = 0.214 (active self: *M* = 223 ms, *SEM* = 18 ms; passive self: *M* = 185 ms, *SEM* = 17 ms), and “other” trials, *F*(1, 22) = 23.599, *p* < .001, *ω*_*p*_^2^ = 0.485 (active other: *M* = 235 ms, *SEM* = 17 ms; passive other: *M* = 182 ms, *SEM* = 21 ms; see also Figure 3b). Note that an internal setup delay of 43 ms must be added to all threshold mean values. Neither the main effect of hand identity nor the interaction between agency and hand identity were significant, *F*(1, 22) = 0.185, *p* = .671, *ω*_*p*_^2^ = −0.035, and *F*(1, 22) = 0.986, *p* = .332, *ω*_*p*_^2^ = −0.0006, respectively. The analysis of slopes revealed no significant effects, agency, *F*(1, 22) = 0.744, *p* = .398, *ω*_*p*_^2^ = −0.011; hand identity, *F*(1, 22) = 2.541, *p* = .125, *ω*_*p*_^2^ = 0.060; agency × hand identity, *F*(1, 22) = 1.036, *p* = .320, *ω*_*p*_^2^ = 0.001. Additional exploratory analyses on behavioral data from the preparatory session are reported in the Supplement (Video S1).

### 3.2 fMRI results

#### 3.2.1 Main effects of agency and hand identity

**Figure 4:**
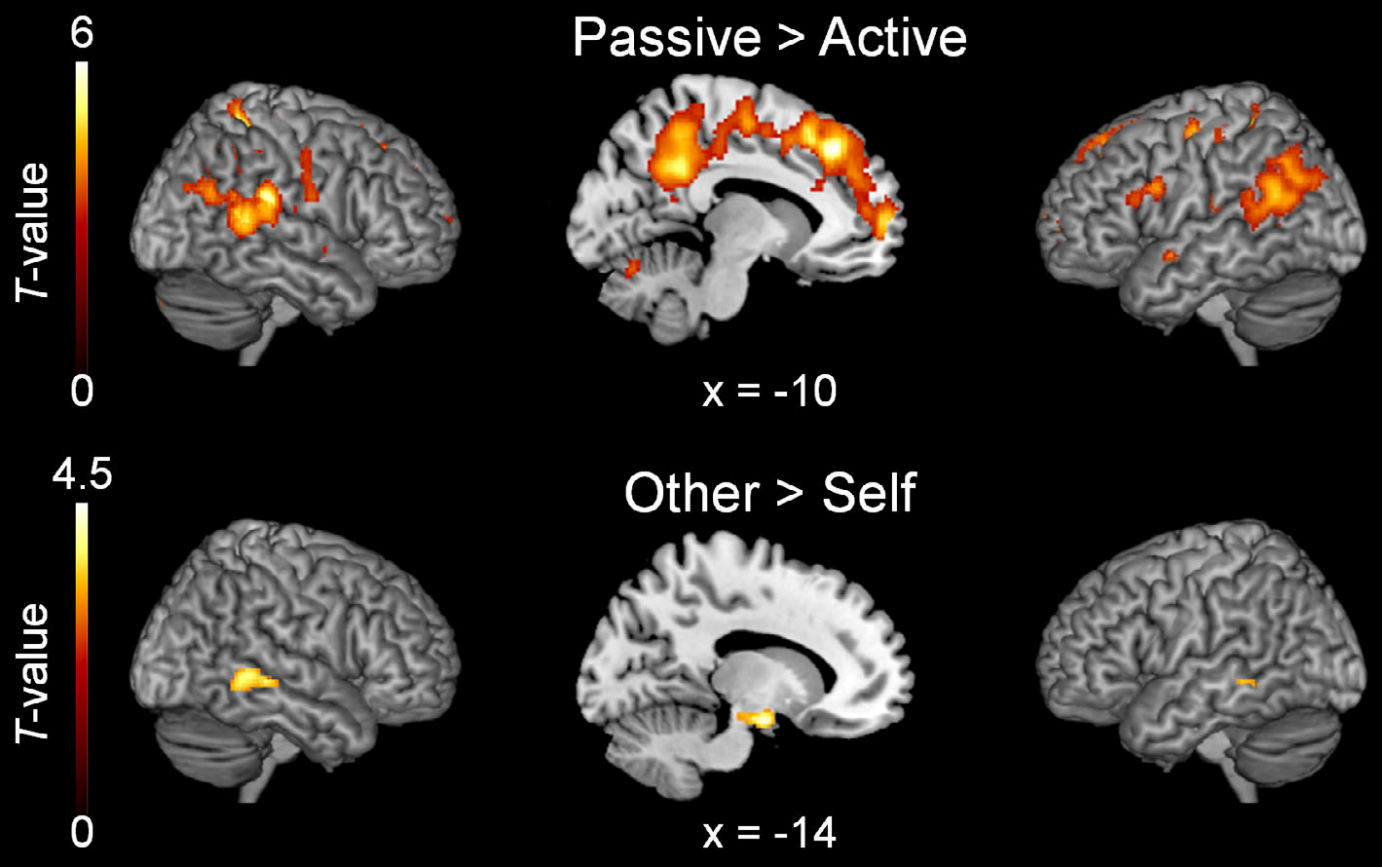
Group level fMRI results (*N* = 23) of main effects. The upper row shows clusters that were less activated during active than passive conditions (main effect of agency). The lower row illustrates brain regions that were more activated during “other” than “self” trials (main effect of hand identity). Cluster defining threshold: *p* < .001, uncorrected. Minimum cluster size = 83 voxels (Monte‐Carlo cluster level corrected at *p* < .05)

Peak activations of the main effects and their locations are displayed in Table 1. To identify brain regions demonstrating reduced activation for the processing of actively generated feedback regardless of hand identity, passive conditions were contrasted with the active conditions, [(passive self + passive other) > (active self + active other)]. This contrast revealed a large cluster with local maxima in the right precuneus, the left superior frontal gyrus, and the right superior parietal lobule (Figure 4, top row; but see also Figure S2). Apart from the local maxima, this cluster also covered areas involved in visual processing (bilateral lingual gyrus, bilateral middle occipital gyrus, bilateral calcarine sulcus, bilateral fusiform gyrus, bilateral superior occipital gyrus, bilateral cuneus, bilateral inferior occipital gyrus, right posterior superior temporal sulcus). Further clusters were found in the right superior temporal gyrus, the lobule VIII of the right cerebellum and the right postcentral gyrus. The opposite contrast [(active self + active other) > (passive self + passive other)] yielded no significant clusters. To investigate whether motor‐related areas (such as the contralateral precentral gyrus) were activated during movement performance (active and passive), we additionally contrasted active [(active self + active other) > baseline] and passive [(passive self + passive other) > baseline] movements against the implicit baseline. This analysis revealed activations in the contralateral precentral gyrus for both active and passive movements (see Table S2 and Figure S3).

**Table 1:** Group level suprathreshold anatomical locations for main effects of agency (active versus passive) and hand identity (“self” versus “other”)

Anatomical locations (local maxima)	Hemisphere	*x*	*y*	*z*	*T*	No. voxels
**Passive > Active**						
Precuneus	Right	18	−48	42	6.79	29,552
Superior frontal gyrus	Left	−8	34	44	6.57	
Superior parietal lobule	Right	18	−46	58	6.41	
Superior temporal gyrus	Right	44	−16	−6	5.51	321
Superior temporal gyrus	Right	52	0	−8	4.12	
Cerebellum lobule VIII	Right	20	−58	−50	4.07	124
Postcentral gyrus	Right	62	−8	22	3.78	178
Postcentral gyrus	Right	62	−10	32	3.65	
Postcentral gyrus	Right	54	−10	36	3.52	
**Active > Passive**						
No significant activations						
**Other > Self**						
Hippocampus	Left	−14	−6	−18	4.40	140
Middle temporal gyrus	Right	64	−42	−2	3.98	179
Middle temporal gyrus	Right	70	−32	−4	3.95	
Middle temporal gyrus	Left	−56	−46	−10	3.73	87
Middle temporal gyrus	Left	−62	−38	−4	3.51	
Inferior temporal gyrus	Left	−52	−54	−14	3.43	
**Self > Other**						
Calcarine sulcus	Right	10	−94	10	6.63	2,815
Lingual gyrus	Left	−6	−66	6	6.10	
Calcarine sulcus	Left	−12	−80	10	5.03	

*Note: N* = 23. Coordinates are listed in MNI space. Indented labels denote local maxima of the cluster extent. Cluster defining threshold: *p* < .001, uncorrected. Minimum cluster size: 83 voxels (Monte‐Carlo cluster level corrected at *p* < .05).

To identify brain regions that were more activated when someone else's compared to the participant's own hand was seen the BOLD signal in “self” conditions was subtracted from “other” conditions, [(active other + passive other) > (active self + passive self)]. This contrast revealed a cluster encompassing the left hippocampus, the left amygdala, and the left parahippocampus. Further clusters were found in the bilateral middle temporal gyrus (Figure 4, bottom row). The opposite contrast [(active self + passive self) > (active other + passive other)] revealed a significant cluster in the bilateral calcarine gyrus, extending to the left lingual gyrus.

#### 3.2.2 Interaction between agency and hand identity

**Figure 5:**
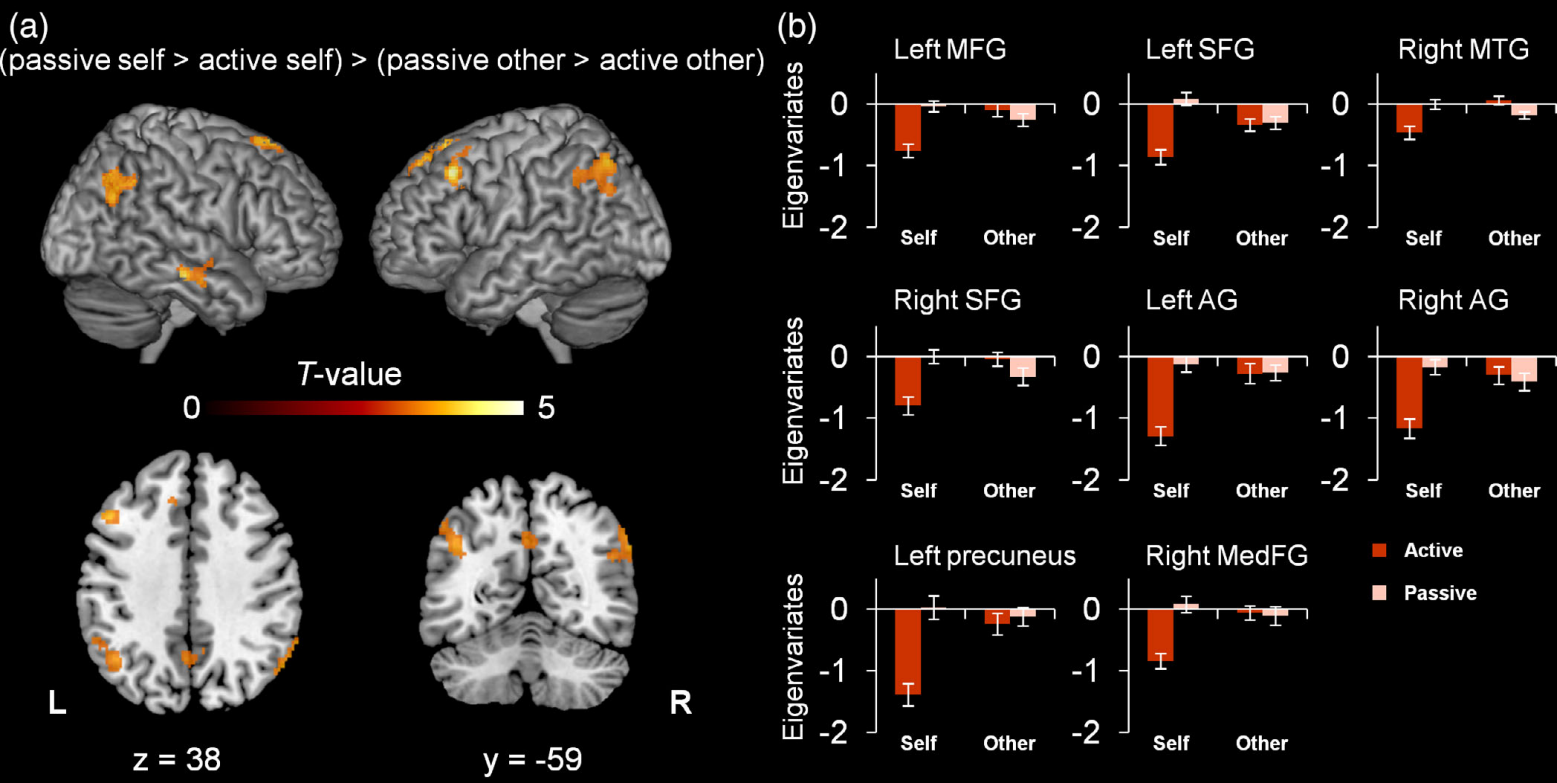
Group level fMRI results (*N* = 23) of the agency × hand identity interaction effect. (a) The clusters represent brain regions where the difference in BOLD‐signal between active and passive conditions was stronger during “self” than “other” trials. Cluster defining threshold: *p* < .001, uncorrected. Minimum cluster size: 83 voxels (Monte‐Carlo cluster level corrected at *p* < .05). L: Left, R: Right. (b) Means of eigenvariates (first principal components) extracted from the corresponding clusters ± standard errors of the means (*SEM*s). AG, angular gyrus; MedFG, medial frontal gyrus; MFG, middle frontal gyrus; MTG, middle temporal gyrus; SFG, superior frontal gyrus

To investigate the interaction between agency and hand identity on the neural level, we identified clusters in which the difference in BOLD signal between passive and active movements was more pronounced when one's own hand was seen, [(passive self > active self) > (passive other > active other)]. The contrast revealed several supra‐threshold clusters in parietal, frontal and temporal cortices (see Table 2 and Figure 5). More specifically, parietal regions encompassed the bilateral angular gyrus and the precuneus. Frontal regions included the left middle frontal gyrus, the bilateral superior frontal gyrus, and the right medial frontal gyrus. Finally, activation in the temporal lobule was found in the right middle temporal gyrus.

**Table 2:** Group level suprathreshold anatomical locations for the interaction effect between agency and hand identity, showing stronger BOLD suppression for active versus passive movements when one's own hand was displayed, [(passive self > active self) > (passive other > active other)]

Anatomical locations (local maxima)	Hemisphere	*x*	*y*	*z*	*T*	No. voxels
Middle frontal gyrus	Left	−46	24	44	4.71	243
Middle frontal gyrus	Left	−34	22	50	3.71	
Middle frontal gyrus	Left	−38	18	44	3.60	
Superior frontal gyrus	Left	−12	26	60	4.38	269
Superior frontal gyrus	Left	−14	42	48	4.03	
Superior frontal gyrus	Left	−8	34	42	3.49	
Middle temporal gyrus	Right	64	−20	−18	4.17	89
Middle temporal gyrus	Right	64	−10	−20	3.56	
Superior temporal gyrus	Right	64	−4	−10	3.34	
Superior frontal gyrus	Right	10	30	62	3.98	106
Superior frontal gyrus	Right	18	26	62	3.85	
Superior frontal gyrus	Right	14	36	58	3.49	
Angular gyrus	Left	−42	−68	50	3.95	367
Angular gyrus	Left	−44	−60	34	3.67	
Angular gyrus	Left	−50	−70	34	3.48	
Angular gyrus	Right	58	−62	30	3.82	173
Angular gyrus	Right	52	−64	42	3.77	
Inferior parietal lobule	Right	62	−50	38	3.68	
Precuneus	Left	−2	−56	34	3.75	155
Precuneus	Left	0	−64	42	3.60	
Precuneus	Right	8	−54	36	3.43	
Medial frontal gyrus	Right	4	54	−14	3.71	98

*Notes: N* = 23. Coordinates are listed in MNI space. Indented labels denote local maxima of the cluster extent. Cluster defining threshold: *p* < .001, uncorrected. Minimum cluster size: 83 voxels (Monte‐Carlo cluster level corrected at *p* < .05).

## 4 DISCUSSION

We investigated whether forward models incorporate the identity of one's own hand when predicting the sensory consequences of voluntary hand movements. On the behavioral level, higher delay detection thresholds during active compared to passive movements indicated suppressed processing during active trials. On a neural level, similar suppression was reflected in reduced BOLD activation in the precuneus, the right superior temporal gyrus, the cerebellum, and the right postcentral gyrus for active compared to passive movements. Furthermore, seeing someone else's versus one's own hand yielded increased activation in the left hippocampus and the bilateral middle temporal gyrus. Most importantly, we found an interaction between agency (active vs. passive) and hand identity (“self” vs.” other”) in parietal (bilateral angular gyrus and precuneus), frontal (left middle frontal gyrus, bilateral superior frontal gyrus, right medial frontal gyrus), and temporal (right middle temporal gyrus) areas. The interaction pattern revealed that BOLD suppression for active compared to passive movements was stronger when participants were viewing their own compared to someone else's hand. These results show for the first time that the brain incorporates hand identity when generating predictions about the sensory consequences of voluntary hand movements.

### 4.1 Behavioral effects

Worse delay detection thresholds for active compared to passive movements indicated that the perception of movement feedback was suppressed during active conditions. According to the forward model framework, perceptual suppression of actively compared to passively generated sensory feedback allows to focus on external stimuli by canceling out predictable sensory input (Cullen, 2004; Pynn & Desouza, 2013; Wolpert & Flanagan, 2001). Since the processing of sensory information of both action and feedback is suppressed during active movements, it is likely that the temporal comparison between action and feedback is affected as well. While the behavioral effects in the current experiment replicate previous results showing worse delay detection performances for actively compared to passively generated movement feedback (Arikan et al., 2019; Pazen et al., 2020; van Kemenade et al., 2019), it contradicts studies reporting enhanced detection performances during active versus passive movements (Schmalenbach, Billino, Kircher, van Kemenade, & Straube, 2017; Shimada, Qi, & Hiraki, 2010; van Kemenade et al., 2016). Importantly, studies showing enhancement effects have used button presses producing briefly displayed action outcomes presented at the end of the movement. In contrast, in studies showing suppression effects (including the current study), participants had to perform continuous movements with relatively large trajectories that were continuously displayed on a screen. It has been shown that participants are more sensitive to discrete action‐outcome asynchronies than to movement‐related feedback (David, Skoruppa, Gulberti, Schultz, & Engel, 2016). Moreover, it has been reported that sensory suppression specifically occurs during movement execution (Juravle, Deubel, Tan, & Spence, 2010). Therefore, we speculate that the effect of active versus passive movements on delay detection performances might—at least in part—be dependent on the continuous co‐occurrence of movement and feedback.

### 4.2 Neural effect of passive versus active movements

Contrasting passive against active movements (passive > active) revealed BOLD suppression in multiple regions including the precuneus, the right superior temporal gyrus, the cerebellum, and the right postcentral gyrus. Suppression was also found in areas involved in visual processing, such as occipital cortices and the right posterior superior temporal sulcus. These results are in line with previous studies showing that neural processing is suppressed for actively generated sensory input (Blakemore et al., 1998; Hughes & Waszak, 2011; Straube et al., 2017). Specifically, our findings on areas involved in somatosensory (e.g., postcentral gyrus) and visual perception replicate that the brain suppresses the processing of actively generated continuous movement feedback (Arikan et al., 2019; Limanowski et al., 2018; Pazen et al., 2020).

The opposite contrast (active > passive) yielded no significant clusters at the applied threshold. Even though it is intuitive to expect motor cortices to be more activated during active than passive movements, it has been shown that motor‐related areas can also be activated by passive movements (Arikan et al., 2019; Blakemore et al., 2003; Onishi, 2018; Onishi et al., 2013; Pazen et al., 2020; Sasaki et al., 2017; Weiller et al., 1996). In line with this, both active and passive movements in our study yielded significant activations in motor cortices when contrasted against the implicit baseline. Furthermore, it has been demonstrated that motor‐related areas can be activated by mere observation of an action (Buccino et al., 2001; Hari et al., 1998; Moriuchi et al., 2017; Rizzolatti, Fadiga, Gallese, & Fogassi, 1996), as well as during experimental conditions where agency is impaired (David et al., 2007; Yomogida et al., 2010). Thus, activation of motor areas in the absence of voluntary movements is not uncommon, which may have led to the absence of significant activation differences in the active versus passive contrast.

### 4.3 Neural effect of “other” versus “self” hand feedback

Contrasting “other” against “self” hand feedback revealed a cluster encompassing the left hippocampus, the left amygdala, and the left parahippocampus, as well as clusters in the bilateral middle temporal gyrus. The hippocampus has been shown to be involved in the initial formation of new memories in the short‐term memory (Lepage, Habib, & Tulving, 1998; Wirth et al., 2003; Zeineh, Engel, Thompson, & Bookheimer, 2003). In our study, participants were presented with unfamiliar hands in the “other” condition. Hence, we speculate that increased activation in the hippocampus during “other” versus “self” trials may reflect processes involved in the encoding of perceptual information about the “other” hand. Furthermore, increased activation in the amygdala during “other” than “self” trials might reflect a difference in the emotional context between these conditions (Gongora et al., 2019), given that the “other” hand deviated from what participants would usually see during daily life (i.e., the own hand). However, since we did not expect this result, this interpretation is merely speculative. Finally, activation in the middle temporal gyrus has been reported in studies on the recognition of self and other (Kircher et al., 2000; Kircher et al., 2001; Kruse et al., 2016; Uddin, Kaplan, Molnar‐Szakacs, Zaidel, & Iacoboni, 2005). While previous studies on self‐recognition have mostly used static images of faces or whole bodies, we have presented participants in our study with videos displaying their own or someone else's hand. Thus, along with previous studies on self‐recognition, our results indicate that the middle temporal gyrus is involved in visual self‐other distinction, and that this process is presumably similar for different parts of the body.

### 4.4 Neural interaction between agency and hand identity

Our findings showing stronger suppression for feedback of one's own compared to someone else's hand provide evidence supporting the hypothesis that the brain takes hand identity into account when predicting the sensory consequences of one's own actions. Although we focused on investigating hand identity, these results strongly remind of recent behavioral studies revealing that suppression of actively generated sensory feedback is stronger when participants experience ownership over the hand causing the stimulus (Kilteni & Ehrsson, 2017a). The influence of ownership on the perception of one's own actions has been linked to efference copy‐based predictive mechanisms, implying that internal forward models generate sensory predictions specifically for the embodied hand (Aymerich‐Franch et al., 2016).

Even though in the current study, differences between “self” and “other” were achieved by varying the identity of the presented hands (rather than ownership), we found stronger suppression for feedback of the participant's own compared to someone else's hand in regions that have previously been associated with action‐feedback monitoring, such as the angular gyrus, or — more generally — the temporo‐parietal junction (Farrer et al., 2008). Previous studies have shown that the temporo‐parietal junction is involved in the processing of temporal or spatial mismatches between actions and their sensory consequences (Farrer et al., 2003; Farrer et al., 2008; Farrer & Frith, 2002; Leube et al., 2010; Leube, Knoblich, Erb, Grodd, et al., 2003; Spence et al., 1997; van Kemenade, Arikan, Kircher, & Straube, 2017). However, it has recently been shown that activation in the angular gyrus due to intersensory conflict arises both for actively and passively generated sensory input, suggesting that the angular gyrus is involved in intersensory matching processes, independent of agency (Shimada et al., 2005; Tsakiris, Longo, et al., 2010; van Kemenade et al., 2019). In our study, intersensory conflict may have been caused by inserting temporal delays between proprioceptive signals during action and the visual display of the movement. However, as the proportion of delays was identical in all conditions, intersensory conflict alone cannot explain our findings of stronger neural suppression for feedback of one's own compared to someone else's hand. Interestingly, posterior parietal areas have also be shown to be activated by semantic incongruities between expected and actual sensory input (Yomogida et al., 2010), indicating that matching processes in this area may not be limited to spatiotemporal features of sensory feedback. Similarly, Jakobs et al. (2009) suggested that the temporo‐parietal junction is linked to a more general predictive process, with activation in this area reflecting increased processing load when updating action expectations in the presence of prediction errors.

Moreover, agency and hand identity interacted in frontal areas, that is, the left middle frontal gyrus, the bilateral superior frontal gyrus, and the right medial frontal gyrus. These brain areas are involved in a range of tasks, including violations of causal relationships (Blos, Chatterjee, Kircher, & Straube, 2012; Danek, Öllinger, Fraps, Grothe, & Flanagin, 2015; Parris, Kuhn, Mizon, Benattayallah, & Hodgson, 2009), processing of action‐outcome discrepancies (Backasch et al., 2014; Farrer et al., 2008), reacting to visual cues under conditions of uncertainty (Jakobs et al., 2009), or self‐referential processing (Ebisch & Aleman, 2016; Northoff & Bermpohl, 2004). Collectively, our findings suggest that the brain engages multiple frontal (e.g., middle, superior, and medial frontal gyrus) and parietal (e.g., angular gyrus) regions when predictions about sensory events are in error, for example, when the active movement results in feedback that displays an unfamiliar instead of the own hand. Therefore, we argue that internal forward models can take the identity of one's hand into account when predicting the sensory consequences of an action, thereby producing fewer prediction errors for actively generated feedback displaying one's own hand.

Furthermore, all regions obtained from the interaction contrast (middle, superior, and medial frontal gyrus, angular gyrus, precuneus, and middle temporal gyrus) have been associated with higher‐order social cognition and self‐referential processing like face recognition (Kircher et al., 2000; Kircher et al., 2001; Platek, Keenan, Gallup, & Mohamed, 2004; Taylor et al., 2009), retrieval of memory contents about one's own person or other people (Ebisch & Aleman, 2016; Maddock, Garrett, & Buonocore, 2001; Northoff & Bermpohl, 2004), or perspective taking (Cavanna & Trimble, 2006; Ruby & Decety, 2001; Vogeley et al., 2004). Moreover, the angular gyrus has been considered a core region of the default mode network, which describes a large‐scale brain system activated during rest as well as during tasks involving social cognition (Andrews‐Hanna, Smallwood, & Spreng, 2014; Meyer, 2019). Specifically, the angular gyrus has been reported to be involved in a wide range of processes such as retrieval of autobiographical and conceptual knowledge, inference of other's mental states, or external agency attribution (for a review, see Seghier, 2013). Overall, the angular gyrus has been denoted a cross‐modal hub that, based on prior expectations and knowledge, integrates perception with interpretation in order to give meaning to events (Seghier, 2013; van Kemenade et al., 2017). These findings suggest that stronger neural suppression for feedback of one's own compared to someone else's hand might additionally reflect social‐cognitive processes required to distinguish between self and other. It has been proposed that the attribution of sensory events to one's own action emerges from a multifactorial weighting process in which internal cues like efference copy signals are integrated with additional external cues (Moore & Fletcher, 2012; Synofzik, Vosgerau, & Newen, 2008). In this sense, our data indicate that bottom‐up agency cues, such as efference copy signals during active movements, and top‐down agency cues, such as the possibility of controlling someone else's hand, are integrated to distinguish between self and other. More specifically, actively controlling a non‐embodied unfamiliar hand during “other” trials might have felt odd, as this contradicts common knowledge about how the world works. Moreover, it has been shown that suppression effects can be modulated by prior beliefs of authorship (i.e., the belief that a sensory event is caused by one's action), possibly by affecting the reliability of sensory predictions (Desantis, Weiss, Schütz‐Bosbach, & Waszak, 2012). Collectively, our data indicate that the integration of bottom‐up and top‐down agency cues consume less resources when participants are able to predict the sensory consequences of their action, that is, when they actively move their own hand, thus giving rise to our neural interaction effect.

### 4.5 The role of the interaction between agency and hand identity in self‐other distinction

Interestingly, neither the neural suppression effect in sensory areas nor delay detection performances were significantly modulated by hand identity. Instead, the interaction effect was found in areas linked to higher‐order social‐cognitive processing. These results suggest a distinction between two aspects of agency, namely (a) agency over the movement and (b) agency over the feedback (see also Christensen & Grünbaum, 2018). While agency over the movement concerns the question of who was the author of a sensory event (e.g., “Did I initiate the movement of the hand?”), agency over the feedback is required to determine whether actively generated feedback actually belongs to one's self (e.g., “Does the moving hand belong to my body?”). For instance, when grabbing and moving another person's hand, hand identity as well as anatomical constraints (e.g., the fact that humans only have one right hand) make it possible to distinguish feedback that is merely generated by one's action (e.g., movement of the “other” hand) from feedback that actually involves one's self (e.g., movement of one's own hand).

We argue that separating these two aspects of agency allows highly efficient sensory processing in social situations: While the brain is able to suppress processing of actively generated (predictable) sensory input, reliable self‐other distinction is still possible. In line with this, our data indicate that agency over the movement might be dependent on comparisons between basic sensory predictions and actual outcomes involving proprioceptive signals (reflected in neural suppression in the postcentral gyrus) and visual signals giving information about, for example, movement trajectories (reflected in neural suppression in visual cortices and the right posterior superior temporal sulcus). Accordingly, behavioral differences in the delay detection task for active versus passive movements could be explained by generally reduced sensory processing, independent of hand identity. In contrast, agency over the feedback also takes hand identity into account, thereby enabling agents to distinguish feedback involving their bodies from other sensory input. The separation between these two aspects of agency might ultimately contribute to efficient sensory processing even when using artificial objects, such as tools or prostheses (Kilteni & Ehrsson, 2017b), which should be further investigated in future studies.

## 5 LIMITATIONS

The study has some limitations that need to be addressed. First, since the feedback in the “self” condition consisted of live recordings of the participant's hand, there was greater interindividual variance in stimulus material during “self” than “other” trials (e.g., participants' clothing was visible in some cases). However, our behavioral data show that delay detection performances did not differ for “self” versus “other” trials, suggesting that participant‐specific visual cues did not influence delay detection performances. Moreover, in the functional data analysis, we compared whether “self” versus “other” trials differed with regard to differences between active and passive conditions (i.e., agency × hand identity interaction effect). Therefore, activation differences between “self” and “other” trials were canceled out. Second, we have no data about the experience of ownership in our task. Thus, our results can only be indirectly related to studies examining the interplay of body ownership and sensory suppression (e.g., Aymerich‐Franch et al., 2016; Burin et al., 2017; Kilteni & Ehrsson, 2017a; Pyasik et al., 2019). However, even though we manipulated hand identity (rather than ownership), we found an interaction in areas previously linked to action‐feedback processing and self‐other distinction. Third, there were only 24 trials per condition in the current study (as also used in Pazen et al., 2020). Despite the small number of trials in the current study, we observed perceptual suppression during active versus passive movements on a behavioral and neural level as well as stronger BOLD suppression for feedback of one's own compared to someone else's hand in areas related to error processing and self‐referential processing. Thus, we are convinced that the number of trials was sufficient for our study purposes. Finally, we want to note that alternative explanations for suppression effects have been proposed (for reviews, see Dogge, Custers, & Aarts, 2019; Hughes, Desantis, Waszak, & Hinshaw, 2013). For instance, it has been shown that temporal prediction (i.e., the ability to predict when a stimulus will occur) and identity prediction (i.e., the ability to predict what stimulus will occur) can be sufficient to produce suppression effects (Hughes et al., 2013; Lange, 2009). However, since delays were randomly inserted between movements (active and passive) and feedback, temporal prediction alone cannot explain the suppression effects observed in the current study. Moreover, visual movement feedback (i.e., displays of the participant's own or the “other” hand) was always presented immediately after the cue so that participants knew which hand they would control. Thus, identity prediction according to Hughes et al. (2013) cannot explain our suppression effects, either. Furthermore, some studies have pointed to possible contributions of postdicitve mechanisms to sensory suppression: Stimuli externally applied to one's body shortly before movement onset are masked by reafferent sensations during ensuing movement execution (Voss, Ingram, Wolpert, & Haggard, 2008; Williams & Chapman, 2002; but see also Bays et al., 2006). However, using direct movement feedback in the current study (i.e., displays of one's own or someone else's hand moving in accordance with one's action), suppression effects were observed by comparing highly controlled active and passive movement conditions. This contradicts postdictive frameworks, according to which sensory processing should be similarly affected by active and passive movements (Voss et al., 2008). Therefore, we argue that efference copy‐based predictions during active movements are the most suitable framework for explaining suppression effects in our data.

## 6 CONCLUSIONS

The current study demonstrated on the neural level that the perception of actively generated hand movements is modulated by the identity of the moving hand. We found that actively compared to passively generated feedback was associated with BOLD suppression in a large cortical network and related to worse delay detection performances. Furthermore, processing of hand identity was related to increased BOLD activation in the left hippocampus and the bilateral middle temporal gyrus for feedback displaying an unfamiliar versus one's own hand. Most importantly, our results revealed an interaction effect between agency and hand identity in posterior parietal (angular gyrus and precuneus), frontal (middle, superior, and medial frontal gyrus) and temporal (middle temporal gyrus) areas. The pattern of this interaction effect indicated that suppression of actively generated feedback was stronger when participants saw their own compared to someone else's hand, suggesting that internal forward models take the identity of the moving body part into consideration when predicting the sensory consequences during the performance of an action. These findings might ultimately advance our understanding of how predictive mechanisms shape self‐other distinction in healthy populations, such as during joint action. The underlying neural mechanisms could further be targeted in the therapy of symptoms associated with disturbed self‐other processing, such as in patients with schizophrenia.

## Supporting information


**Appendix** **S1**. Supplement.

**Video S1**. Supporting Information

## Data Availability

The data that support the findings of this study are openly available in Zenodo at: http://doi.org/10.5281/zenodo.2621302.
