# Supplementary material for: Seeing your own or someone else's hand moving in accordance with your action: The neural interaction of agency and hand identity
Source: Hum Brain Mapp. 2020 Feb 24;41(9):2474–89. doi: 10.1002/hbm.24958 (PMC7268012; doi:10.1002/hbm.24958)
Supplement: Supplementary file 1 — Appendix S1. Supplement. [file HBM-41-2474-s001.docx]

Supplement

1. Post-experiment questionnaire

To check whether the manipulation of hand identity was successful, participants were handed out a questionnaire after the experiment. All questions on hand identity and mean response values are listed in Supplementary Table 1.

| **Supplementary Table 1.** Post-experiment hand identity questionnaire. | | | |
| --- | --- | --- | --- |
|  | Question | Possible answers | Group means ± standard errors of the means (SEMs) |
| Q1a | How much did you feel that you were seeing your own hand during trials with your own hand? | 1 (very weak) to 10 (very strong) | 7.61 ± 0.52 |
| Q1b | How much did you feel that you were seeing your own hand during trials with the other hand? | 1 (very weak) to 10 (very strong) | 4.83 ± 0.60 |
| Q2 | As how similar did you perceive your own and the other hand? | 1 (very dissimilar) to 10 (very similar) | 2.74 ± 0.42 |
| Q3 | How difficult was it to distinguish your own hand from the other hand? | 1 (very easy) to 10 (very difficult) | 1.70 ± 0.25 |

2. Results from the preparatory session

For the sake of completeness, delay detection performance in the preparatory session was explored as well. Using psignifit4, psychometric functions were fit to response data for each condition. Detection thresholds and slopes were derived from the functions and entered into separate factorial repeated-measures ANOVAs. The analysis of thresholds revealed no significant effects, agency, *F*(1, 22) = 1.737, *p* = .201, *ω*_p_² = 0.029; hand identity, *F*(1, 22) = 0.769, *p* = .390, *ω*_p_² < 0; agency × hand identity, *F*(1, 22) = 1.941, *p* = .177, *ω*_p_² = 0.038. Similarly, no significant effects were observed in terms of the slopes, agency, *F*(1, 22) = 0.868, *p* = .362, *ω*_p_² < 0; hand identity, *F*(1, 22) = 2.142, *p* = .157, *ω*_p_² = 0.089; agency × hand identity, *F*(1, 22) = 1.880, *p* = .184, *ω*_p_² = 0.035. However, in line with the behavioral data from the fMRI session, threshold values indicated that detection performance in the preparatory session was descriptively worse during active (*M* = 200 ms, *SEM* = 17 ms) than passive trials (*M* = 184 ms, *SEM* = 15 ms; Supplementary Figure 1). Therefore, we argue that the low number of trials in the preparatory session can explain the lack of a significant effect of agency on detection thresholds, although we cannot rule out potential training effects.


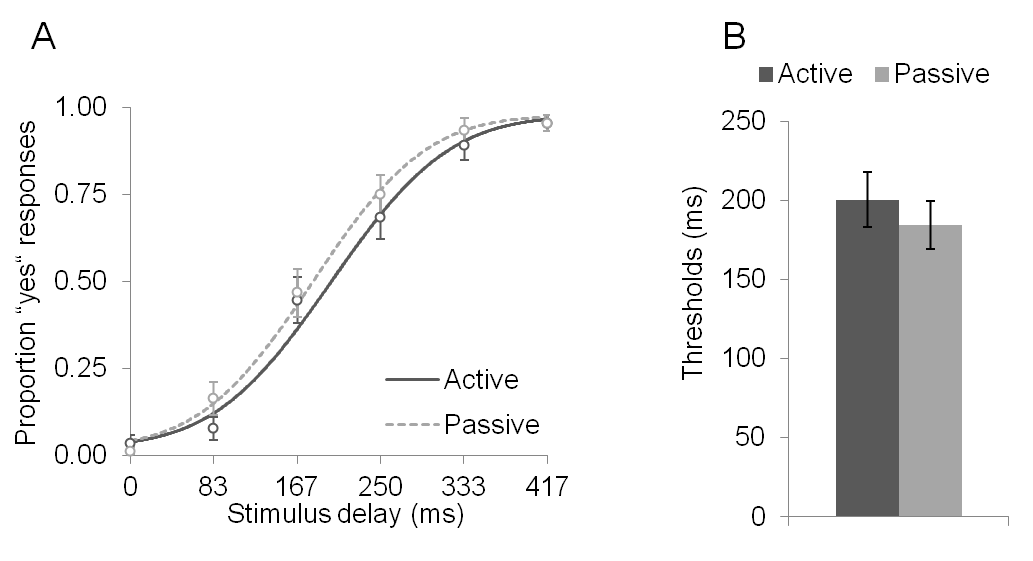


**Supplementary Figure 1.** Delay detection performance during active and passive conditions in the preparatory session. (A) Group psychometric functions (*N* = 23). (B) Mean thresholds (delays at which the probability for “yes” responses regarding the presence of a delay was 0.5, based on the fit psychometric function). Error bars show ± standard errors of the means (SEMs).

3. Overlap between regions activated by main effects and the interaction effect

We checked whether regions activated by the interaction contrast [(passive self > active self) > (passive other > active other)] overlapped with regions activated by the main effect of agency (passive > active). There was overlap in the left superior frontal gyrus (90%), the left precuneus (86%), the left angular gyrus (67%), the right medial frontal gyrus (36%), the right angular gyrus (29%), the left middle frontal gyrus (9%), and the right superior frontal gyrus (3%; percentage values refer to the amount of overlap in clusters activated by the interaction contrast; see Supplementary Figure 2). Since the pattern of the interaction effect indicated that suppression in these regions strongly differed between “self” and “other” feedback, the main effect of agency (passive > active) must be interpreted with caution in the overlapping regions. There was no overlap between regions from the interaction contrast and the other > self contrast.


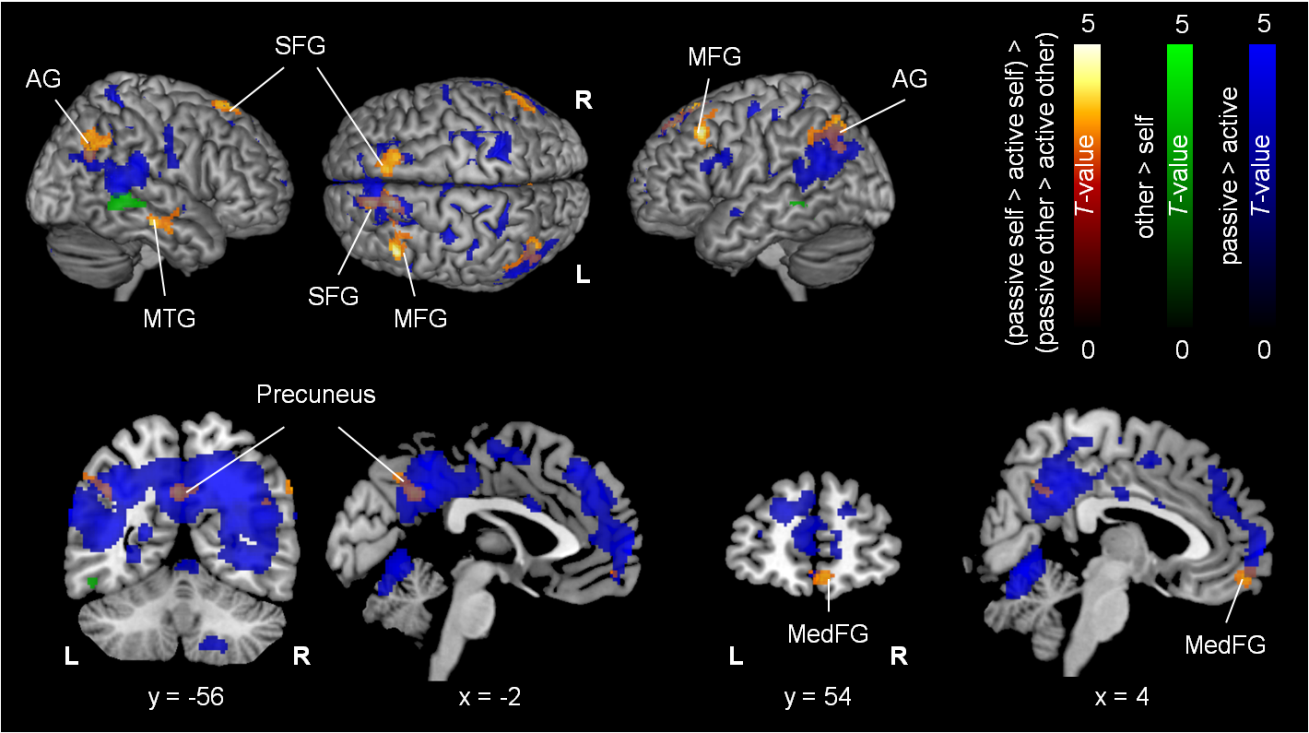


**Supplementary Figure 2.** Overlap between regions activated by the interaction contrast [(passive self > active self) > (passive other > active other)] and the main effects of hand identity (other > self) and agency (passive > active). L: Left, R: Right. AG: Angular gyrus, MedFG: Medial frontal gyrus, MFG: Middle frontal gyrus, MTG: Middle temporal gyrus, SFG: Superior frontal gyrus. Cluster defining threshold: *p* < .001, uncorrected. Minimum cluster size = 83 voxels (Monte-Carlo cluster level corrected at *p* < .05).

4. Baseline contrasts

To check whether motor-related areas (e.g., contralateral precentral gyrus) were activated during movement execution, baseline contrasts were calculated for active (active > baseline) and passive (passive > baseline) conditions. Both contrasts revealed activations in the contralateral precentral gyrus (see Supplementary Table 2 and Supplementary Figure 3)

| **Supplementary Table 2** Group level suprathreshold anatomical locations of baseline contrasts for active (active > baseline) and passive (passive > baseline) conditions. | | | | | | | |
| --- | --- | --- | --- | --- | --- | --- | --- |
| Anatomical Locations  (Local Maxima) |  | Hemisphere | x | y | z | *T* | No. voxels |
| **Active > baseline** |  |  |  |  |  |  |  |
| Middle occipital gyrus |  | Left | -22 | -98 | 10 | 13.42 | 17699 |
| Middle occipital gyrus |  | Left | -44 | -76 | -2 | 13.41 |  |
| Superior occipital gyrus |  | Left | -12 | -96 | 4 | 13.24 |  |
| Postcentral gyrus |  | Left | -54 | -22 | 50 | 13.25 | 5641 |
| Precentral gyrus |  | Left | -42 | -22 | 64 | 12.77 |  |
| Precentral gyrus |  | Left | -28 | -22 | 74 | 11.64 |  |
| Precentral gyrus |  | Right | 48 | 8 | 30 | 7.93 | 1942 |
| Inferior frontal gyrus |  | Right | 50 | 10 | 18 | 6.61 |  |
| Middle frontal gyrus |  | Right | 44 | -2 | 56 | 5.92 |  |
| Thalamus |  | Left | -20 | -28 | -2 | 7.64 | 924 |
| Thalamus |  | Right | 20 | -28 | 0 | 6.42 |  |
| Thalamus |  | Left | -6 | -20 | -10 | 4.40 |  |
| Insula |  | Left | -40 | -2 | 12 | 6.83 | 271 |
| Rolandic operculum |  | Right | 48 | -22 | 20 | 6.36 | 1443 |
| Postcentral gyrus |  | Right | 48 | -22 | 20 | 6.28 |  |
| Inferior parietal lobule |  | Right | 34 | -44 | 48 | 5.08 |  |
| Precentral gyrus |  | Left | -60 | 8 | 26 | 4.93 | 192 |
| Precentral gyrus |  | Left | -52 | 2 | 46 | 4.42 |  |
| Precentral gyrus |  | Left | -58 | 8 | 34 | 4.17 |  |
| Insula |  | Left | -34 | 16 | 6 | 4.25 | 112 |
| Insula |  | Left | -28 | 26 | 0 | 3.83 |  |
|  |  |  |  |  |  |  |  |
| **Passive > baseline** |  |  |  |  |  |  |  |
| Postcentral gyrus |  | Left | -52 | -22 | 52 | 15.81 | 11493 |
| Postcentral gyrus |  | Left | -46 | -26 | 60 | 15.66 |  |
| Precentral gyrus |  | Left | -40 | -12 | 62 | 12.95 |  |
| Middle temporal gyrus |  | Right | 46 | -70 | -2 | 14.99 | 20814 |
| Middle occipital gyrus |  | Left | -48 | -72 | 2 | 14.67 |  |
| Calcarine sulcus |  | Left | -10 | -92 | 0 | 14.02 |  |
| Superior temporal gyrus |  | Right | 60 | -32 | 22 | 9.17 | 7209 |
| Rolandic operculum |  | Right | 46 | -26 | 22 | 8.83 |  |
| Inferior frontal gyrus |  | Right | 50 | 8 | 28 | 8.57 |  |
| Thalamus |  | Left | -14 | -20 | 6 | 7.71 | 898 |
| Thalamus |  | Left | -18 | -28 | 0 | 7.49 |  |
| Pallidum |  | Left | -22 | -8 | 4 | 4.22 |  |
| Insula |  | Right | 34 | 24 | 4 | 6.87 | 405 |
| Thalamus |  | Right | 18 | -28 | 2 | 5.97 | 373 |
| Thalamus |  | Right | 12 | -18 | 6 | 5.15 |  |
| *Note: N* = 23. Coordinates are listed in MNI space. Indented labels denote local maxima of the cluster extent. Cluster defining threshold: *p* < .001, uncorrected. Minimum cluster size: 83 voxels (Monte-Carlo cluster level corrected at *p* < .05). | | | | | | | |


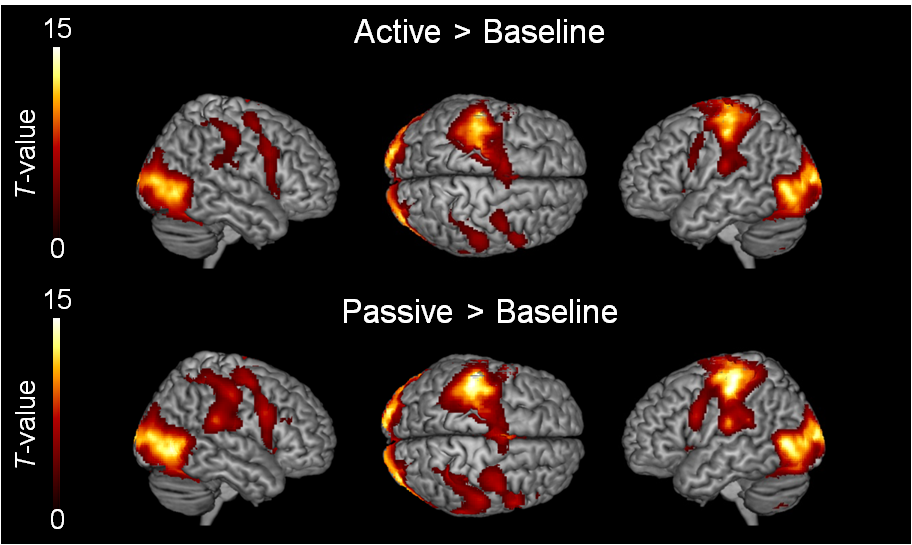
**Supplementary Figure 3.** Group level fMRI results (*N* = 23) of baseline effects for active and passive conditions. Cluster defining threshold: *p* < .001, uncorrected. Minimum cluster size = 83 voxels (Monte-Carlo cluster level corrected at *p* < .05).
